# Identification of *Glycyrrhiza* as the rikkunshito constituent with the highest antagonistic potential on heterologously expressed 5-HT_3A_ receptors due to the action of flavonoids

**DOI:** 10.3389/fphar.2015.00130

**Published:** 2015-07-03

**Authors:** Robin Herbrechter, Paul M. Ziemba, Katrin M. Hoffmann, Hanns Hatt, Markus Werner, Günter Gisselmann

**Affiliations:** Department of Cell Physiology, Ruhr-University BochumBochum, Germany

**Keywords:** rikkunshito, *Glycyrrhiza*, flavonoids, hesperetin, (-)-liquiritigenin, glabridin, licochalcone A, 5-HT_3A_ receptor

## Abstract

The traditional Japanese phytomedicine rikkunshito is traditionally used for the treatment of gastrointestinal motility disorders, cachexia and nausea. These effects indicate 5-HT_3_ receptor antagonism, due to the involvement of these receptors in such pathophysiological processes. E.g., setrons, specific 5-HT_3_ receptor antagonists are the strongest antiemetics, developed so far. Therefore, the antagonistic effects of the eight rikkunshito constituents at heterologously expressed 5-HT_3A_receptors were analyzed using the two-electrode voltage-clamp technique. The results indicate that tinctures from *Aurantii*, *Ginseng*, *Zingiberis*, *Atractylodis* and *Glycyrrhiza* inhibited the 5-HT_3A_ receptor response, whereas the tinctures of *Poria cocos*, *Jujubae* and *Pinellia* exhibited no effect. Surprisingly, the strongest antagonism was found for *Glycyrrhiza*, whereas the *Zingiberis* tincture, which is considered to be primarily responsible for the effect of rikkunshito, exhibited the weakest antagonism of 5-HT_3A_ receptors. Rikkunshito contains various vanilloids, ginsenosides and flavonoids, a portion of which show an antagonistic effect on 5-HT_3_ receptors. A screening of the established ingredients of the active rikkunshito constituents and related substances lead to the identification of new antagonists within the class of flavonoids. The flavonoids (-)-liquiritigenin, glabridin and licochalcone A from *Glycyrrhiza* species were found to be the most effective inhibitors of the 5-HT-induced currents in the screening. The flavonoids (-)-liquiritigenin and hesperetin from *Aurantii* inhibited the receptor response in a non-competitive manner, whereas glabridin and licochalcone A exhibited a potential competitive antagonism. Furthermore, licochalcone A acts as a partial antagonist of 5-HT_3A_ receptors. Thus, this study reveals new 5-HT_3A_ receptor antagonists with the aid of increasing the comprehension of the complex effects of rikkunshito.

## Introduction

The 5-HT_3_ receptor channels are the only ionotropic receptors within the 5-HT receptor family and belong to the cys-loop family of ligand-gated ion channels (Derkach et al., 1989; Hannon and Hoyer, 2008). These channels occur in the PNS and are highly expressed in the trigeminus (Manteniotis et al., 2013) and the enteric nervous system (Niesler et al., 2003). In the CNS, they are expressed in the striatum, substantia nigra, amygdala, hippocampus and nucleus accumbens (Boess and Martin, 1994). Other 5-HT_3_ receptor expressing structures are the nucleus tractus solitarius and the area postrema (Boess and Martin, 1994), which are parts of the vomiting center that trigger nausea and vomiting.

5-HT_3_ receptors are involved in many pathophysiological processes, such as nociception and gastrointestinal motility disorders, and the development of nausea and vomiting, therefore showing broad clinical relevance (Doak and Sawynok, 1997; Gershon, 2004; Jeggo et al., 2005; Costedio et al., 2007). Specific 5-HT_3_ receptor antagonists, such as ondansetron, are mainly used for the treatment of nausea in various conditions, such as chemotherapy-induced nausea and vomiting (CINV), and nausea during the postoperative phase (PONV) (Cubeddu et al., 1994; Gyermek, 1995).

Many 5-HT_3_ receptor antagonists have been identified to date. In addition to the competitively acting setrons (Hope et al., 1996), non-competitive antagonists have been identified within the family of cannabinoids, such as Δ^9^-THC, anandamide and cannabidiol (Barann et al., 2002). Moreover, steroids (Barann et al., 1999) and the anesthetics ketamine, propofol, methohexital and pentobarbital (Barann et al., 1993, 2000a,b, 2008) have been shown to antagonize 5-HT_3_ receptors. Many plant compounds also act as 5-HT_3_ receptor antagonists. For example, alkaloids, such as nicotine (Schreiner et al., 2014), hot substances and terpenes, e.g., bilobalide and ginkgolide B (Thompson et al., 2011), gingerols (Walstab et al., 2013) and many others, were reviewed by Walstab et al. (2010).

Kampo is a traditional Japanese phytomedicine that has its seeds in traditional Chinese medicine. Rikkunshito, a combination of eight constituents, is one of the most famous and prescribed kampo medicines (Tominaga and Arakawa, 2015), and it has a well-known physiological effect on the gastrointestinal system, shows orexigen and antiemetic effects, and takes part in the regulation of peristalsis (Takeda et al., 2008; Tominaga et al., 2011; Yanai et al., 2013; Fujitsuka and Uezono, 2014). These effects indicate an antagonism of 5-HT_3_ receptors. Therefore, the antagonistic effects of the eight constituents of rikkunshito (*Aurantii* pericarpium, *Ginseng* radix, *Zingiberis* rhizoma, *Jujubae* (*Zizyphi*) frucutus, *Pinellia* tuber, *Atractylodis* rhizoma, *Glycyrrhiza* radix and *Poria cocos* (Hoelen) were investigated as ethanol tinctures. Furthermore, we investigated the established ingredients of the active rikkunshito constituents to identify new 5-HT_3A_ receptor antagonists. Although the antagonistic and hence the antiemetic effect of *Ginseng* and *Zingiberis* due to the action of ginsenosides, gingerols and shogaols is well-described (Ernst and Pittler, 2000; Kim et al., 2005; Lee et al., 2007; Haniadka et al., 2012; Ding et al., 2013), there is currently little knowledge of the effect of the residual rikkunshito constituents on 5-HT_3_ receptors.

The aim of this study was the evaluation of the relative contribution of the single constituents of rikkunshito to 5-HT_3_ receptor antagonism and the identification of new antagonists. Therefore, we tested the modulatory effect of tinctures and single substances on heterologously expressed human 5-HT_3A_ receptors using the two-electrode voltage-clamp technique. Surprisingly, *Glycyrrhiza* was identified as the most effective antagonistic tincture among the rikkunshito constituents. Therefore, we concentrated on the investigation of *Glycyrrhiza* ingredients and identified several new flavonoids as 5-HT_3A_ receptor antagonists. The drug Radix *Glycyrrhiza* is used in Kampo medicine for the treatment of pain, gastric ulcers and inflammations of the gastrointestinal and respiratory systems due to its antiphlogistic effect (Kim et al., 2008). A contribution of Radix *Glycyrrhiza* to the antiemetic effect of rikkunshito due to the action of flavonoids is conceivable.

## Materials and methods

### Expression system

The expression plasmid contains the cDNA coding for the 5-HT_3A_ protein in pcDNA3 (Invitrogen) (Lobitz et al., 2001). cRNAs were prepared using the AmpliCap T7 high-yield message marker kit (Epicenter, Madison, WI, USA), following the manufacturer's protocol. *Xenopus laevis* oocytes were obtained as previously described (Sherkheli et al., 2010) and injected with a total amount of 7–20 ng of the receptor-coding cRNA using an injection-setup from WPI (Nanoliter 2000, Micro4). The injected oocytes were stored in ND 96 (96.0 mM NaCl, 2.0 mM KCl, 1.8 mM CaCl_2_, 1.0 mM MgCl_2_, 5.0 mM HEPES, pH 7.2, 200 U/ml penicillin, and 200 μg/ml streptomycin) at 17°C. Measurements were performed one to 5 days after cRNA injection.

### Electrophysiology

The electrophysiological recordings were performed using the two-electrode voltage-clamp technique as previously described (Saras et al., 2008). All of the measurements were performed in normal frog ringer (NFR) [115 mM NaCl, 2.5 mM KCl, 1.8 mM CaCl_2_, 10 mM HEPES; pH 7.2 (NaOH/HCl)] containing niflumic acid (NA) (100 μM) to block the Ca^2+^-induced currents mediated by the intrinsic chloride channel (TMEM16A) or under Ca^2+^-free conditions [115 mM NaCl, 2.5 mM KCl, 1.8 mM MgCl_2_, 10 mM HEPES; pH 7.2 (NaOH/HCl)]. All of the substances were applied after preincubation (30 s). The currents were recorded at a holding potential of typically −60 mV using the Cell Works 6.1.1. software (NPI).

### Solvent controls

To exclude any unrequested effects of the solvents ethanol and DMSO, we tested their direct activation on non-injected and 5-HT_3A_ receptor-expressing oocytes. At the maximal used concentration (1 Vol.-%), a negligible direct activation was observed. Moreover, the modulatory effect on the 5-HT_3A_ receptor response was tested at concentrations of 1.0 Vol.-% for ethanol and DMSO. Ethanol exhibited an inhibition of 14.1 ± 2.6%, and DMSO exhibited an inhibition of 29.1 ± 4.7% (*n* = 6−11). Equivalent volumes of ethanol and DMSO were added to the reference 5-HT solutions. To resolve glycyrrhizin, the solution had to be acidified (pH 5.5). Therefore, we checked the modulatory effect of the pH values on 5-HT_3A_ receptors. Low extracellular pH values inhibited the currents but high pH showed potentiating effects (Supplementary Figure 2).

### Action of the tinctures on non-injected *Xenopus* oocytes

In the control experiments, at a concentration of 1 Vol.-%, the tinctures of *Ginseng*, *Zingiberis* and *Atractylodis* evoked currents in some non-injected oocytes with desensitizing responses (data not shown). In our experiments with 5-HT_3A_ receptor-expressing cells, oocytes were rejected if the amplitude of this direct activation was greater than 10% of the 5-HT-induced current; thus, the direct action of the extracts could not prevent the identification of pronounced blocking effects. Moreover, in our blocking experiments, these currents were desensitized during the 30-s preincubation with the tincture. At a concentration of 0.1 Vol.-%, none of the tinctures evoked any current different from that obtained from the control application of Ringer‘s solution.

### Evaluation of competitive and non-competitive antagonists

To determine the apparent mode of antagonism of the identified tinctures and substances with antagonistic effects, we tested the inhibition of currents induced by low (2.5 μM) and high (30 μM) 5-HT concentrations. In the case of a non-competitive mechanism, the inhibition should be independent of the 5-HT concentration, whereas the efficacy of competitive antagonists decreases with increasing 5-HT concentrations. Alternatively to a competitive mechanism, the dependence of the inhibition on the agonist concentration can also be caused by an allosteric modulation. As a control, we tested the competitive antagonists ondansetron (1 nM) and d-tubocurarine (20 μM) (Hope et al., 1996) and the non-competitive antagonist picrotoxin (50 μM) (Das and Dillon, 2005). As expected, the inhibition of picrotoxin was independent of the 5-HT concentration, and the inhibition obtained with d-tubocurarine and ondansetron was reduced at higher 5-HT concentrations (Table 1). Thus, the method used may indicate the mode of antagonism. Nevertheless, a definitive differentiation between competitive, allosteric, and concentration-dependent antagonists must be performed using ligand binding assays.

**Table 1:** **Competitive and non-competitive action of the identified antagonists and tinctures with antagonistic effect**.

**Tincture/Substance**	**Concentration**	**Mean inhibition ± SEM [%]**		**Significance level/mode of antagonism**
		**5-HT [2.5 μM]**	**5-HT [30 μM]**	
**CONTROLS**				
Picrotoxin	50 μM	72.5 ± 3.7	73.0 ± 2.8	ns/nc
D-tubocurarine	20 μM	82.0 ± 3.5	45.9 ± 4.8	^**^/c
Ondansetron	1 nM	82.4 ± 3.4	57.9 ± 5.3	^*^/c
**TINCTURES**				
*Zingiberis*	1 Vol.-%	20.1 ± 4.9	22.2 ± 4.2	ns/nc
*Aurantii*	1 Vol.-%	37.3 ± 5.4	28.8 ± 2.6	ns/nc
*Ginseng*	1 Vol.-%	44.6 ± 8.7	36.6 ± 7.3	ns/nc
*Atractylodis*	1 Vol.-%	47.1 ± 5.6	27.4 ± 9.1	^*^/c
*Glycyrrhiza*	1 Vol.-%	64.5 ± 9.7	69.1 ± 7.4	ns/nc
Unsweetened licorice	1 Vol.-%	44.6 ± 5.7	13.7 ± 6.6	^***^/c
**SUBSTANCES**				
Atractylenolide III	1 mM	55.0 ± 6.2	57.8 ± 6.0	ns/nc
Licochalcone A	1 mM	76.1 ± 6.1	58.7 ± 2.6	^*^/c
Glabridin	100 μM	64.7 ± 2.1	17.3 ± 2.3	^***^/c
Hesperetin	1 mM	77.3 ± 9.0	79.7 ±6.1	ns/nc
(-)-liquiritigenin	0.5 mM	84.5 ± 2.4	93.5 ± 0.5	^*^/nc

The inhibitions of the 5-HT_3A_ receptor responses by the controls (picrotoxin, ondansetron and d-tubocurarine) and different tinctures and substances at 5-HT concentrations of 2.5 and 30 μM are shown. The level of significance between the inhibitions obtained with two different 5-HT concentrations is indicated by asterisks in the last column (ns, not significant). Furthermore, the apparent mode of antagonism is listed in the last column (c, apparently competitive; nc, non-competitive) (n = 5–7).

### Tinctures and substances

Ethanol tinctures of the rikkunshito constituents were obtained from Dr. Peter Lepke (Kronen Apotheke Wuppertal, Germany). Thus, plant preparations at appropriate quality for Japanese kampo medicine were extracted [200 g crushed plant material in 1 l ethanol (45–90% v/v)] for 10 days at room temperature. The tinctures were obtained by filtrating the supernatant, therefore containing no large solid parts of the plants. The dry weight of the extracted substances was determined by removing the solvent under vacuum (Supplementary Table 2). We used the following tinctures in our study:

*Aurantii* Pericarpium [*Citrus reticulata* Blanco (Rutaceae)] (chinpi, Chen Pi), White *Ginseng* Radix [*Panax ginseng* C.A.Mey. (Araliaceae)] (ninjin, Ren Shen), *Zingiberis* viridis Rhizoma [*Zingiber officinale* Roscoe (Zingiberaceae)] (*shôkyô*, Sheng Jiang), *Jujubae* Fructus [*Ziziphus jujuba* Mill. (Rhamnaceae)] (*taisô*, Da Zao), *Pinelliae* Tuber (*Pinellia ternata* [Thunb.) Makino (Araceae)] (hange, Ban Xia), *Atractylodis macrocephala* Rhizoma [*Atractylodes macrocephala* Koidz. (Asteraceae)] (*sôjutsu*, Bai Zhu), *Glycyrrhiza* Radix [*Glycyrrhiza uralensis* Fisch. (Fabaceae)] (*kanzô*, Gan Cao) and *Poria cocos* [*Wolfiporia extensa* (Peck) Ginns (Polyporaceae)] (*bukuryô*, Fu Ling).

Unsweetened licorice (*Liquirizia purissima* from R. De Rosa, Italy) was inlayed in ethanol (70% v/v) under the same conditions. The chemicals were obtained from Sigma Aldrich (5-HT hydrochloride, niflumic acid (blocker for Ca^2+^ activated chloride channels), picrotoxin (non-competitive ion channel blocker), d-tubocurarine (competitive nACh and 5-HT_3_ receptor antagonist), ondansetron (specific, competitive 5-HT_3_ receptor antagonist), (-)-liquiritigenin, licochalcone A, hesperidin, hesperetin, glabridin and glycyrrhizin), Carl Roth (rutin) and PhytoLab (atractylenolide III). The substances were diluted in water, dimethyl sulfoxide (DMSO) or ethanol.

### Data analysis

The test substances were applied in an alternating manner with 5-HT. Therefore, the currents of the test substances or the modulated currents were normalized to the mean of the 5-HT-induced currents before and after the test substance was applied. The concentration-response data were fitted with the Hill equation with variable slope using SigmaPlot 8.0 (SPSS). Thereby, the calculation of the EC_50_ and IC_50_-values was done. The deviations are represented by the standard error of the mean (SEM). The datasets were tested for statistically significant differences through Student's *t*-test using Excel 2010 (Microsoft) (^*^p < 0.05; ^**^p < 0.005; ^***^p < 0.0005). For multiple comparisons, the significance levels were corrected via Bonferroni-correction.

## Results

### Effect of tinctures of rikkunshito constituents

**Figure 1:**
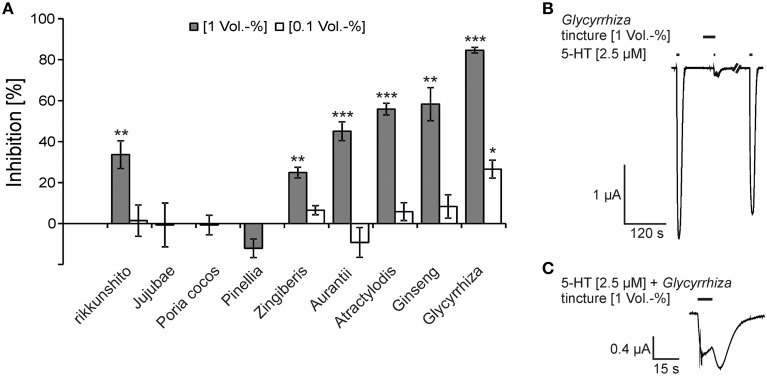
**Inhibition of 5-HT_3A_ receptors by tinctures of rikkunshito and its constituents (A) and original traces from the inhibition by the** ***Glycyrrhiza*** **tincture (B,C)**. **(A)** The dark bars represent the inhibition at 1 Vol.-%, and the bright bars represent that at a concentration of 0.1 Vol.-%. The inhibition from left to right are as follows: 33.7 ± 6.8%, 1.5 ± 7.6%, −0.7 ± 10.7%, −0.7 ± 4.8%, −12.0 ± 4.5%, 24.9 ± 2.6%, 6.5 ± 2.2%, 45.1 ± 4.6%, −9.2 ± 7.3%, 55.9 ± 2.8%, 5.8 ± 4.3%, 58.3 ± 8.1%; 8.4 ± 5.7%, 84.6 ± 1.4% and 26.6 ± 4.4% (*n* = 5 − 8) (^*^p < 0.05; ^**^p < 0.005; ^***^p < 0.0005). **(B,C)** The time of application is denoted by the application bars above the trace. The inhibition was reversible after a 150-s washout (holding potential = −60 mV). **(C)** The 5-HT-induced currents showed a characteristic rebound when co-applied with the *Glycyrrhiza* tincture.

The gastroprokinetic and antiemetic effects of rikkunshito (Tominaga et al., 2011) could be explained by an antagonism of 5-HT_3A_ receptors. To test this hypothesis, we tested the modulatory effects of the constituents of rikkunshito on the 5-HT_3A_ responses. In the first experiments, we tested the effect of the respective tinctures to provide guidance for the identification of the most effective constituents. Under our experimental conditions an EC_50_ value for 5-HT of 2.39 ± 0.06 μM was determined for the 5-HT_3A_ receptor (Supplementary Figure 1). This EC_50_ value is similar to previously reported EC_50_ values evaluated in *Xenopus* oocytes (about 2 μM 5-HT) (Lee et al., 2008; Schreiner et al., 2014). The tinctures from rikkunshito and its constituents *Aurantii*, *Ginseng*, *Zingiberis*, *Atractylodis*, and *Glycyrrhiza*, inhibited the 5-HT_3A_ receptor responses (5-HT 2.5 μM, approximately EC_50_) at a concentration of 1 Vol.-%, whereas the tinctures of *Poria cocos*, *Jujubae* and *Pinellia* exhibited no effect (Figure 1A). Among the five tinctures that exerted an inhibitory effect, the tincture of *Glycyrrhiza* showed the strongest inhibition (84.6 ± 1.4%) (Figure 1B), and the weakest effect was obtained with the *Zingiberis* tincture (24.9 ± 2.6%). The inhibition obtained with the rikkunshito tincture (33.7 ± 1.5%) was close to that calculated by the addition of the effects of the tinctures of the eight constituents with regard to their mass distribution in the decoction (27.5%) (Supplementary Table 1). The 5-HT_3A_ receptor responses showed a huge rebound when co-applied with *Glycyrrhiza* (Figure 1C). All of the six inhibitory tinctures were also tested at a lower concentration (0.1 Vol.-%). In these experiments, only the tincture of *Glycyrrhiza* showed a significant inhibition (26.6 ± 4.4%) (Figure 1A). All of the inhibitions were reversible after a 150-s washout.

### Modulatory effect of the rikkunshito ingredients and tincture of unsweetened licorice

The tinctures contain many different chemical substances. To identify the active compounds, we assessed the modulatory effects of some known ingredients of these plants on 5-HT_3A_ receptors. We focused on the ingredients of *Glycyrrhiza uralensis* that showed the strongest antagonism within the rikkunshito constituents and other ingredients of further *Glycyrrhiza* species. Thus, we tested a tincture of unsweetened licorice because it is obtained from *Glycyrrhiza glabra* L. (Fabaceae). We used a concentration of 1 mM for all of the substances with the exception of glabridin (100 μM) and 1 Vol-% for the licorice tincture.

**Figure 2:**
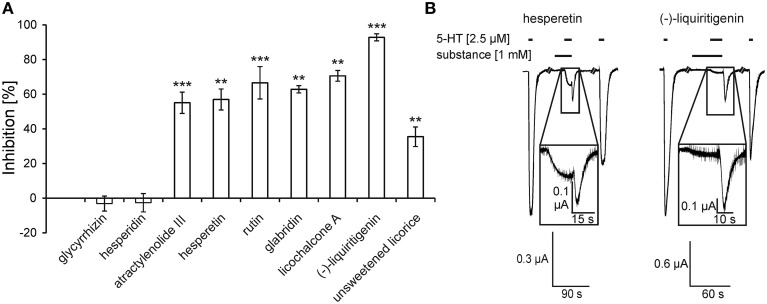
**5-HT_3A_ receptor inhibition by the substances and the unsweetened licorice tincture (A) and original traces of the antagonistic effect of hesperetin and (-)-liquiritigenin (B)**. **(A)** Of the tested substances, the flavonoids exhibited the strongest inhibition with hesperetin and rutin from *Aurantii* and licochalcone A and (-)-liquiritigenin from *Glycyrrhiza* species. The inhibition from left to right are the following: −3.1 ± 4.3%, −2.6 ± 5.3%, 55.0 ± 6.2%, 57.0 ± 6.1%, 66.6 ± 9.3%, 62.8 ± 2.1%, 70.6 ± 3.1%, 92.8 ± 2.0%, and 35.5 ± 5.7% (*n* = 5 − 9) [tincture = 1 Vol.-%, substances = 1 mM except glabridin (100 μM)] (^**^p < 0.005; ^***^p < 0.0005). **(B)** The time of application is denoted by the application bars above the traces. The inhibition was reversible after a 150-s washout. The 5-HT-induced currents showed a characteristic rebound when co-applied with hesperetin or (-)-liquiritigenin (magnifications).

Four ingredients of *Glycyrrhiza* species were investigated. Glycyrrhizin and the flavonoid (-)-liquiritigenin are ubiquitous within the plants of the genus *Glycyrrhiza*, whereas the flavonoids glabridin and licochalcone A are restricted to *G. glabra* and *G. inflata* and *G. eurycarpa* (Xu et al., 1997; Rauchensteiner et al., 2005; Kondo et al., 2007). Glycyrrhizin exhibited no modulatory effect (−3.1 ± 4.3%), whereas glabridin revealed an inhibition of 62.8 ± 2.1%. Licochalcone A and (-)-liquiritigenin were the most effective antagonists, showing inhibitions of 70.6 ± 3.1% and 92.8 ± 2.2%, respectively (Figure 2A). Other flavonoids found in *Aurantii*, namely hesperetin and rutin, also exhibited antagonistic effects. Hesperidin, the most abundant ingredient in rikkunshito (Tominaga et al., 2011), revealed no effect. The flavonoids hesperetin and (-)-liquiritigenin caused 5-HT-induced responses with huge rebounds, similar to those obtained with the Radix *Glycyrrhiza uralensis* tincture (compare Figures 1C, 2B). The tincture of the unsweetened licorice caused an inhibition of 5-HT-induced currents of 35.5 ± 5.7%.

### Competitive and non-competitive action of the identified antagonists

The tinctures of *Atractylodis* and unsweetened licorice, as well as glabridin and licochalcone A, exhibited an apparent competitive antagonism. The tincture of *Zingiberis and Glycyrrhiza* and (-)-liquiritigenin showed increased inhibition at the higher agonist concentration (30 μM), indicating a non-competitive blocking mechanism (Table 1).

### Concentration-inhibition curves of the identified antagonists

**Figure 3:**
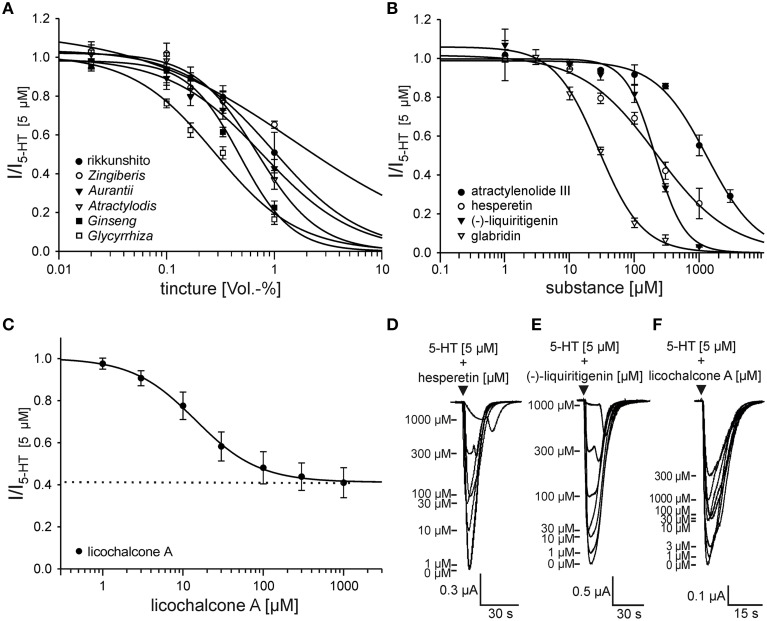
**Concentration-inhibition curves of the antagonistic rikkunshito tinctures (A), substances (B,C) and original traces from the recordings of hesperetin (D), (-)-liquiritigenin (E) and licochalcone A (F)**. **(A)** The calculated IC_50_ values for the tinctures are the following: 1.025 ± 0.2 Vol.-% for rikkunshito (•), 1.517 ± 0.869 Vol.-% for *Zingiberis* (o), 0.774 ± 0.096 Vol.-% for *Aurantii* (▼), 0.685 ± 0.028 Vol.-% for *Atractylodis* (∇), 0.481 ± 0.046 Vol.-% for *Ginseng* (■) and 0.281 ± 0.051 Vol.-% for *Glycyrrhiza* (□) (*n* = 4 − 6). **(B)** The calculated IC_50_ values for the substances are the following: 1322 ± 145 μM for atractylenolide III (•), 218 ± 38 μM for hesperetin (o), 210 ± 25 μM for (-)-liquiritigenin (▼) and 28.0 ± 2.8 μM for glabridin (∇) (*n* = 5 − 7). **(C)** The inhibition by licochalcone A stagnated at 59.0 ± 7.1%, resulting in a residual current of approximately 40% remaining (dashed line). The IC_50_ value was 14.4 ± 1.3 μM (*n* = 5 − 8). **(D–F)** Application of 5-HT + inhibitor (▼) leads to currents, whose maximal amplitude (labeled with -) decreases with increasing concentrations of the inhibitor. The inhibition obtained with licochalcone A stagnated above a concentration of 10 μM, whereas the inhibition induced by hesperetin and (-)-liquiritigenin showing rebounds at concentrations greater than 300 μM and increased with increases in the concentrations until nearly the whole response was blocked.

To characterize and quantify the action of the identified antagonists, we generated concentration-inhibition curves using 5 μM 5-HT. The tinctures inhibited the 5-HT_3A_ receptor responses with regard to their IC_50_ values expressed as Vol.-% in the following order: *Glycyrrhiza* (0.281 ± 0.051) > *Ginseng* (0.481 ± 0.046) > *Atractylodis* (0.685 ± 0.028) > *Aurantii* (0.774 ± 0.096) > *Zingiberis* (1.517 ± 0.869) (Figure 3A). This ranking order was identical to that obtained previously (Figure 1A). The potency of the flavonoids hesperetin (IC_50_ = 218 ± 38 μM) and (-)-liquiritigenin (IC_50_ = 210 ± 25 μM) were similar (Figure 3B). The inhibition by licochalcone A stagnated at approximately 60%, indicating that it shows characteristics of a partial antagonist. In addition to glabridin (IC_50_ = 28.0 ± 2.8 μM), licochalcone A showed the highest potency (IC_50_ = 14.4 ± 1.3 μM) (Figures 3B,C).

## Discussion

Rikkunshito is a Japanese herbal medicine that shows orexigen and antiemetic effects (Takeda et al., 2008; Fujitsuka and Uezono, 2014; Tominaga and Arakawa, 2015). Moreover, it is involved in the regulation of peristalsis and digestion (Tominaga et al., 2011) and therefore ameliorates symptoms of functional dyspepsia and irritable bowel syndrome (Oka et al., 2014). These effects and the antiemetic properties of rikkunshito may be explained by 5-HT_3_ receptor antagonism. Therefore, we assessed the modulatory effect of the eight rikkunshito constituents as ethanol tinctures to identify the most effective constituents and to find new specific antagonists for the 5-HT_3A_ receptor.

We detected an antagonistic effect on 5-HT_3A_ receptors exerted by the tinctures of rikkunshito, *Aurantii*, *Ginseng*, *Zingiberis*, *Atractylodis*, and *Glycyrrhiza*. Surprisingly, *Zingiberis*, which was initially thought to be mainly responsible for the effects of rikkunshito, was the weakest identified antagonist. However, we identified Radix *Glycyrrhiza uralensis* as the strongest 5-HT_3A_ receptor antagonist among the tinctures (Figures 1A, 3A), presumably through the action of (-)-liquiritigenin.

The flavonoid glycoside hesperidin from *Aurantii* is one of the most abundant flavonoids in rikkunshito. It shows a gastroprokinetic effect similar to that of the specific 5-HT_3_ receptor antagonist ondansetron, suggesting that hesperidin is a 5-HT_3_ receptor antagonist (Tominaga et al., 2011). However, in our experiments, only hesperetin, the aglycone of hesperidin, exhibited 5-HT_3A_ receptor antagonism. Tominaga et al. used an *in vivo* animal experimental paradigm in which hesperidin was applied orally. Therefore, it is possible that hesperidin is converted into the active substance by deglycosylation *in vivo.* The lack of 5-HT_3A_ receptor antagonism for hesperidin may be explained by steric problems caused by glycosylation with the disaccharide rutinose. However, other glycosylated flavonoids are known, e.g., rutin from *Aurantii*, which shows an antagonistic effect on 5-HT_3_ receptors. However, also in this case, quercetin, the aglycone of rutin, is the more potent substance (Lee et al., 2008).

The tincture of *Atractylodis*, which is used in kampo medicine for the treatment of nausea and cachexia, showed a strong, apparently competitive antagonism. In our study, atractylenolide III, a weak antagonist (IC_50_ = 1322 ± 145 μM), was the only ingredient that was investigated. However, investigations of other ingredients, such as atractylol, atractylon or biatractylolid (Shao et al., 2014), could lead to the identification of competitive 5-HT_3A_ receptor antagonists with higher potency.

Strong 5-HT_3A_ receptor antagonism was also observed for the tincture of *Ginseng*, which is used due to its antiemetic effect. Kim et al. showed that *Ginseng* extracts reduce cisplatin-induced nausea in ferrets (Kim et al., 2005). Steroid glycosides, called ginsenosides, are accountable for the observed 5-HT_3A_ receptor antagonism (Min et al., 2003; Lee et al., 2007), and their binding site in the pore region of 5-HT_3A_ receptors has been identified (Lee et al., 2007).

The vanilloids gingerol and shogaol (Abdel-Aziz et al., 2005, 2006; Walstab et al., 2013) as well as the diterpene lactone galanolactone (Huang et al., 1991) are responsible for the antagonistic effect of the *Zingiberis* tincture. Abdel-Aziz et al. showed that gingerols and shogaols inhibit the contractions of isolated guinea pig and rat ilea induced by a specific 5-HT_3_ receptor agonist (Abdel-Aziz et al., 2005, 2006), indicating 5-HT_3_ receptor antagonism for the spasmolytic effects of *Zingiberis* and rikkunshito. Our study supports the proposal of these vanilloids as the active principles of *Zingiberis* due to the non-competitive antagonism of this tincture, which was previously reported for gingerol and shogaol (Walstab et al., 2013). In many clinical trials, *Zingiberis* was able to reduce nausea under various conditions, such as motion sickness, *hyperemesis gravidarum*, CINV and PONV (Ernst and Pittler, 2000; Haniadka et al., 2012; Ding et al., 2013).

**Figure 4:**
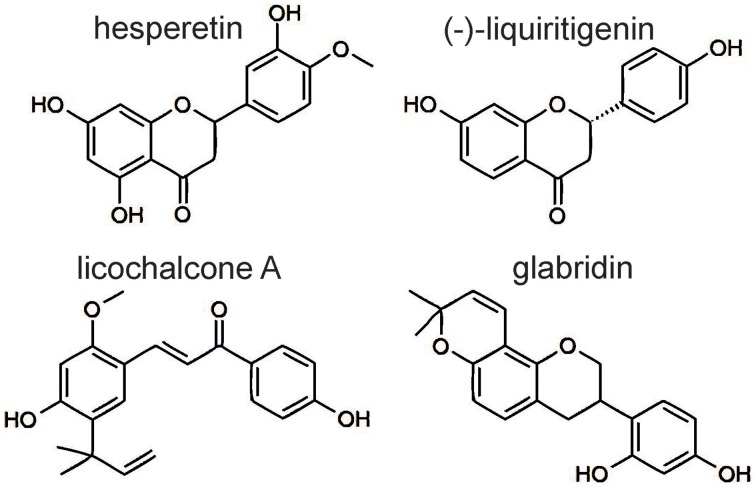
**Chemical structure of the investigated flavonoids**. Hesperetin and (-)-liquiritigenin share similar molecular structures.

Radix *Glycyrrhiza* is used for the treatment of gastric ulcer and inflammations of the gastrointestinal and respiratory system (Kim et al., 2008). Among the rikkunshito constituents, *Glycyrrhiza uralensis* is the antagonistic tincture with the highest efficacy and potency. Moreover, we tested a tincture of unsweetened licorice, which is sourced from *Glycyrrhiza glabra* and usually consumed. This tincture was also able to inhibit the 5-HT_3A_ receptor responses. We tested four *Glycyrrhiza* ingredients, namely the glycoside glycyrrhizin and the flavonoids glabridin, (-)-liquiritigenin and licochalcone A. Although (-)-liquiritigenin and glycyrrhizin are ubiquitous within the plants of the genus *Glycyrrhiza*, glabridin and licochalcone A are restricted to specific species. Glabridin is a specific ingredient of *Glycyrrhiza glabra*, and licochalcone A is detectable only in the species *G. inflata* and *G. eurycarpa* (Xu et al., 1997; Rauchensteiner et al., 2005; Kondo et al., 2007). Glycyrrhizin, which is used for the treatment of epilepsy, chronic gastritis and obstipation (Hänsel et al., 1993), showed no 5-HT_3A_ receptor antagonism. The flavonoids glabridin, (-)-liquiritigenin and licochalcone A exhibited the strongest antagonism among our tested substances. (-)-Liquiritigenin appears to be the active component in *Glycyrrhiza uralensis*. Both showed non-competitive antagonism and lead to a huge rebound of the inhibited 5-HT_3A_ receptor responses. In addition to (-)-liquiritigenin, hesperetin exhibited similar kinetics at high concentrations (Figures 3E,F). Because both flavonoids are chemically related (Figure 4), possess similar IC_50_ values and share a non-competitive antagonism, a common binding site at the receptor is hypothesized.

Kim et al. investigated the antiphlogistic effect of liquiritigenin and attributed this effect to the inhibition of NF-_K_B, an important transcription factor in the immune response (Kim et al., 2008). Therefore, liquiritigenin may contribute to the antiemetic and antiphlogistic effects of rikkunshito. In contrast, (-)-liquiritigenin cannot be accountable for the concentration-dependent 5-HT_3A_ receptor antagonism of the licorice tincture. Instead, the apparently competitive blocker glabridin, which is a potentiator of the closely related GABA_A_ receptor (Jin et al., 2013), may be the main antagonist of the unsweetened licorice, which is sourced from *G. glabra*. The blocker licochalcone A differs from hesperetin and (-)-liquiritigenin by its chemical structure and its action as a partial antagonist (Figures 3D–F, 4). The combination of concentration-dependency and partial blocker properties is unusual. Although these properties allude to the action of licochalcone A as a partial agonist, this consideration can be ruled out due to the absence of the direct activation of 5-HT_3A_ receptors. The hypothesis of a concentration-dependent, allosteric antagonist whose maximal inhibition decreases with increasing agonist concentrations is more likely.

We showed that the *Glycyrrhiza uralensis* tincture exhibits the strongest inhibition of 5-HT_3A_ receptor responses compared with the rest of the rikkunshito constituents, possibly due to the action of the flavonoid (-)-liquiritigenin. Other *Glycyrrhiza* species share this flavonoid-based 5-HT_3A_ receptor antagonism, which is attributable to the antagonists glabridin and licochalcone A, a partial 5-HT_3A_ receptor antagonist. These results contribute to a better understanding of the action of rikkunshito at a pharmacological level and allow the establishment of flavonoids as a new potent class of plant ingredients with regard to 5-HT_3_ receptor antagonism. Therefore, flavonoids appear to be at least equally active antagonists as gingerols and shogaols from *Zingiberis* and ginsenosides from *Ginseng*, which were thought to be responsible for the antiemetic properties of rikkunshito prior to this study. However, it should be mentioned, that the identified antagonists are inferior to already commonly used drugs like setrons due to their lower potency. Hence, a comparatively higher concentration of flavonoids must be reached to cause physiologically relevant effects. If those concentrations of substances with 5-HT_3_ receptor antagonism can be reached by rikkunshito under naturally-occuring conditions is hard to define. However, rikkunshito contains flavonoids, ginsenosides, and vanilloids, three well-investigated classes of plant ingredients that inhibit 5-HT_3A_ receptors by binding to the receptor, presumably at independent binding sites. Hence, a synergistic drug interaction with additional or maybe mutual potentiating character is conceivable. In an exemplary experiment, we demonstrated that the combined action of distinct plant derived blockers can lead to an increased block of the 5-HT_3A_ receptor (Supplementary Figure 3). In addition to that, a contribution of structurally related, unemployed substances, e.g., liquiritin and its apiosides and glycosides to the 5-HT_3_ receptor antagonism seems to be likely. In this study, we refuted the assumption that hesperidin, the main ingredient of rikkunshito, promotes gastric emptying via 5-HT_3_ receptor antagonism due to the lack of antagonism obtained in our screening. Instead, hesperetin, the aglycone of hesperidin, acts as a 5-HT_3A_ receptor antagonist. Nevertheless, a 5-HT_3_ receptor mediated effect of hesperidin is assumed due to the occurrence of deglycosylation *in vivo*. This study contributes to a better understanding of the action of rikkunshito at a pharmacological level and emphasizes the importance of *Glycyrrhiza* and *Aurantii* for the antagonism of 5-HT_3A_ receptors.

### Conflict of interest statement

The authors declare that the research was conducted in the absence of any commercial or financial relationships that could be construed as a potential conflict of interest.
