# Supplementary material for: Identification of Glycyrrhiza as the rikkunshito constituent with the highest antagonistic potential on heterologously expressed 5-HT3A receptors due to the action of flavonoids
Source: Front Pharmacol. 2015 Jul 3;6:130. doi: 10.3389/fphar.2015.00130 (PMC4490227; doi:10.3389/fphar.2015.00130)
Supplement: Supplementary file 1 [file DataSheet1.PDF]

Supplementary Material

Identification of *Glycyrrhiza* as the rikkunshito constituent with the highest antagonistic potential on heterologously expressed 5-HT$_{3A}$ receptors due to the action of flavonoids

Robin Herbrechter, Paul M. Ziemba, Katrin M. Hoffmann, Hanns Hatt, Markus Werner* and Günter Gisselmann*

Affiliation

Department of Cell Physiology, Ruhr-University Bochum, Bochum, Germany

*both authors contributed equally

Correspondence: Günter Gisselmann; Ruhr-University Bochum; Department of Cell Physiology; ND-4/165; Universitätsstraße 150, D-44780 Bochum, Germany

E-mail address: guenter.gisselmann@rub.de

1. Supplementary Tables

Supplementary Table 1. Constituents of rikkunshito (according to Eberhard (2003))

Constituent:	Plant name:	Mass distribution [%]:
Aurantii Pericarpium	*Citrus reticulata* Blanco (Rutaceae)	9.3 %
White *Ginseng* Radix	*Panax ginseng* C.A.Mey. (Araliaceae)	18.6 %
Zingiberis viridis Rhizoma	*Zingiber officinale* Roscoe (Zingiberaceae)	2.3 %
Jujubae Fructus	*Ziziphus jujuba* Mill. (Rhamnaceae)	9.3 %
Pinelliae Tuber	*Pinellia ternata* (Thunb.) Makino (Araceae)	18.6 %
Atractylodis macrocephala Rhizoma*	*Atractylodes macrocephala* Koidz. (Asteraceae)	18.6 %
Glycyrrhiza uralensis Radix	*Glycyrrhiza uralensis* Fisch. (Fabaceae)	4.7 %
Poria cocos	*Wolfiporia extensa* (Peck) Ginns (Polyporaceae)	18.6 %

* instead of *Atractylodis ovata* Rhizoma (*Atractylodes ovata* (Thunb.) DC. (Asteraceae))

Eberhard, U., (2003). *Leitfaden Kampo-Medizin, Japanische Phytotherapie* 1. edition., Munich: Elsevier GmbH.

Supplementary Table 2. Dry weight of the extracted substances in mg per ml tincture

**Constituent:**	**Plant name:**	**Dry weight [mg/ml]:**
Aurantii Pericarpium	*Citrus reticulata* Blanco (Rutaceae)	63.2 mg/ml
White *Ginseng* Radix	*Panax ginseng* C.A.Mey. (Araliaceae)	29.4 mg/ml
Zingiberis viridis Rhizoma	*Zingiber officinale* Roscoe (Zingiberaceae)	11.6 mg/ml
Jujubae Fructus	*Ziziphus jujuba* Mill. (Rhamnaceae)	141.6 mg/ml
Pinelliae Tuber	*Pinellia ternata* (Thunb.) Makino (Araceae)	3.6 mg/ml
Atractylodis macrocephala Rhizoma	*Atractylodes macrocephala* Koidz. (Asteraceae)	56.4 mg/ml
Glycyrrhiza uralensis Radix	*Glycyrrhiza uralensis* Fisch. (Fabaceae)	64.4 mg/ml
Poria cocos	*Wolfiporia extensa* (Peck) Ginns (Polyporaceae)	4.8 mg/ml
Liquirizia purissima	*Glycyrrhiza glabra* L. (Fabaceae)	20.6 mg/ml

2. Supplementary Figures

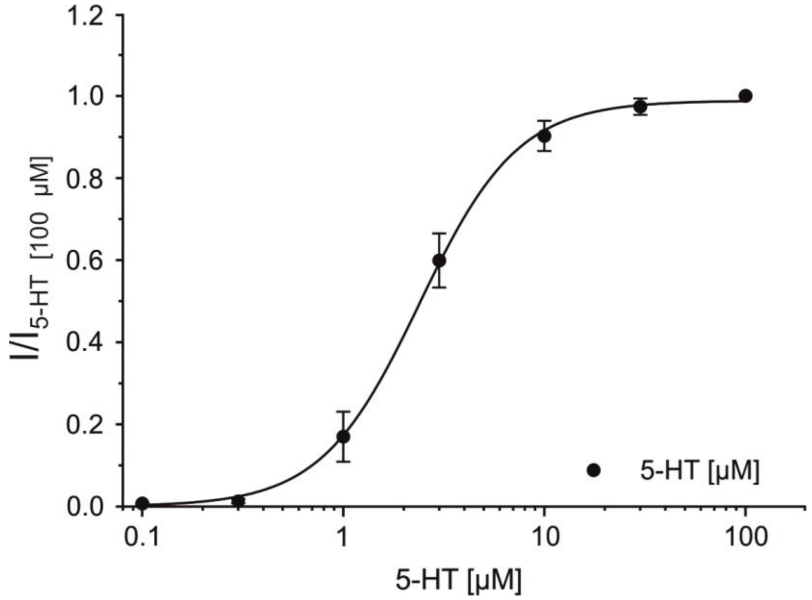

Supplementary Figure 1. Concentration-effect curve of 5-HT on 5-HT$_{3A}$ receptors, heterologously expressed in *Xenopus laevis* oocytes. Current amplitudes are normalized to responses evoked by a saturating 5-HT concentration of 100 µM. The calculated EC$_{50}$ value amounts 2.39 ± 0.06 µM 5-HT (n=8-19).

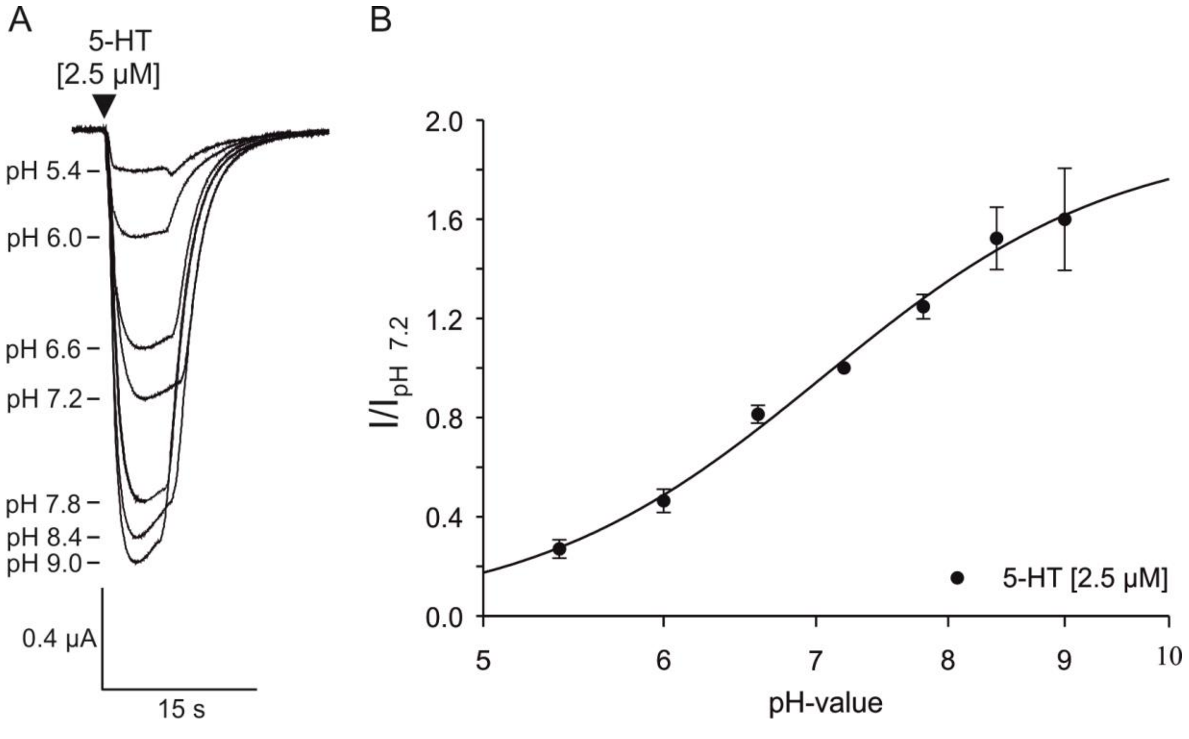

Supplementary Figure 2. Illustration of pH modulation of 5-HT$_{3A}$ receptors by original traces (A) and graphically (B). A) Application of 5-HT [2.5 µM] (▼) leads to currents, the maximal amplitudes of which are labeled as (-). Although low extracellular pH values inhibited the 5-HT$_{3A}$ receptor response, a potentiating effect occurred at pH > 7.2 (holding potential = -60 mV). **B)** Graphical depiction for the modulating effect of the pH value (n = 10).

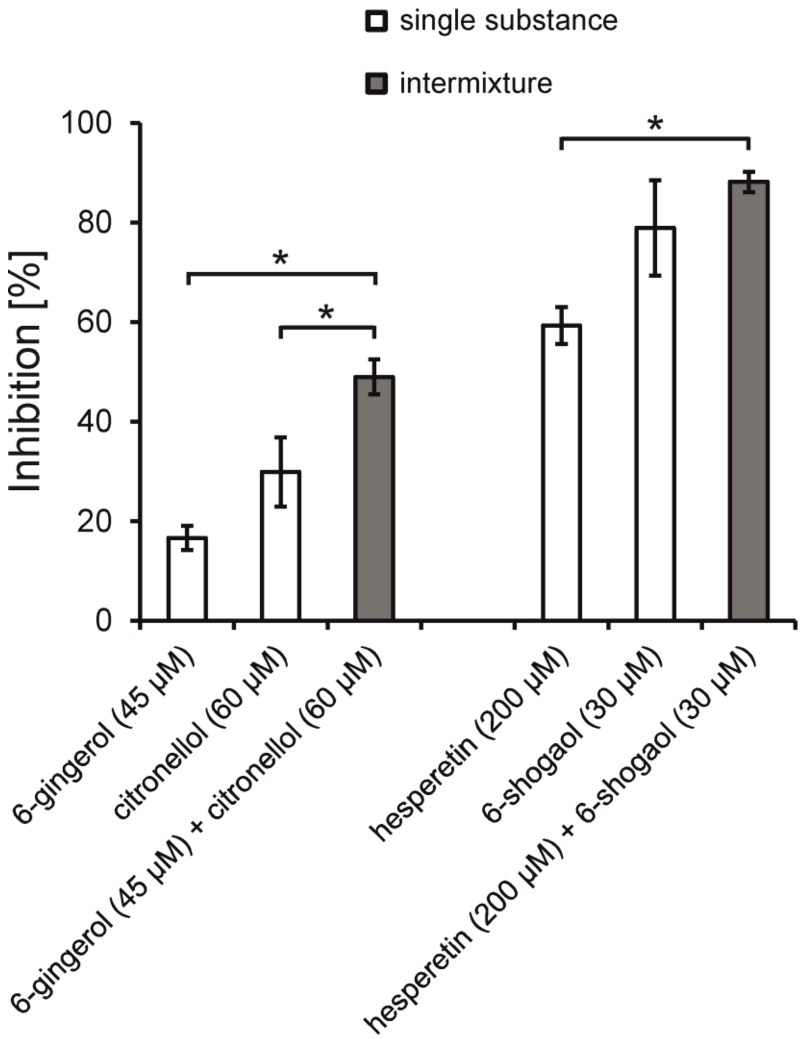

Supplementary Figure 3. Synergistic effects between different herbal 5-HT$_{3A}$ receptor antagonists. The investigated substances 6-gingerol (vanilloid) and citronellol (acyclic monoterpenoid) as well as hesperetin (flavonoid) and 6-shogaol (vanilloid) exhibited synergistic effects. Statistical analysis was done using Student´s t test (n=5-9).
